# Shaping the future of care for patients with Ehlers-Danlos syndromes: from multidisciplinary management to precision medicine

**DOI:** 10.1186/s13023-025-03615-5

**Published:** 2025-03-03

**Authors:** Kexin Xu, Guozhuang Li, Terry Jianguo Zhang, Nan Wu

**Affiliations:** 1 Department of Orthopedic Surgery, State Key Laboratory of Complex Severe and Rare Diseases, Peking Union Medical College Hospital, Chinese Academy of Medical Sciences & Peking Union Medical College, Beijing, 100730 China; 2 Beijing Key Laboratory of Big Data Innovation and Application for Skeletal Health Medical Care, Beijing, 100730 China; 3 Key Laboratory of Big Data for Spinal Deformities, Chinese Academy of Medical Sciences, Beijing, 100730 China

Ehlers-Danlos syndromes (EDS) are a group of hereditary connective tissue disorders (HCTDs) with considerable clinical and genetic heterogeneity. To date, 21 genes have been associated with EDS; however, the genetic basis of the most prevalent subtype—hypermobile EDS—remains unknown [1–3]. Traditionally, EDS is recognized by the classic triad of joint hypermobility, skin hyperextensibility, and tissue fragility. Yet, EDS often leads to disabling or life-threatening multisystemic comorbidities, such as scoliosis, cardiac dysautonomia, vascular dissection or aneurysms, and pneumothorax, which overlap across different subtypes and with other HCTDs. Recent studies have further revealed several continuous phenotypic spectra, including hypermobility spectrum disorders, *COL1*-related overlap disorder, and linkeropathies, involving various EDS subtypes [4–6]. All these complexities pose significant challenges in the diagnosis and treatment of EDS.

As orthopedic surgeons, we are often the first specialists that patients with unrecognized EDS encounter, seeking care for joint dislocations, spinal deformities, and skeletal dysplasia [7–9]. Therefore, it is crucial for us to recognize both skeletal and extra-skeletal features to make a timely and accurate diagnosis, a cornerstone of precision medicine. Worldwide, significant advancements have been made in achieving precision diagnoses, integrating deep phenotyping, family-based genome sequencing, RNA sequencing, and periodic reanalysis to optimize diagnostic yield [10–13]. Recently, the focus has shifted toward developing effective management strategies. With Rare Disease Day 2025 approaching, this editorial will explore current and potential management strategies for EDS, focusing on both skeletal and multisystemic involvement. We aim to promote greater research collaboration across global scientific and clinical communities to accelerate our understanding of EDS and advance the development of targeted, effective management strategies.

## Current management through a multidisciplinary approach

The current management of EDS, like many other rare disorders, is primarily symptomatic and has largely depended on the physician’s experience. It is not uncommon for patients with EDS to consult various healthcare professionals across different departments and institutions. In order to provide better care for patients and their families, the first guidelines on the diagnosis and management of EDS have been established [14, 15]. Meanwhile, emerging studies suggest more effective evidence-based management options, providing insights into the most prominent challenges specific to various EDS subtypes, such as pain in hypermobile EDS (hEDS), wound care in classic EDS (cEDS), and vasculopathy in vascular EDS (vEDS).

## Evolving management approaches informed by molecular findings

In recent years, scientific exchange on EDS has increased unprecedentedly, with a growing focus on molecular finding-guided management strategies. Surgical planning and long-term monitoring of vascular comorbidities in vEDS are gradually shifting toward genotype-guided approaches. For example, variants leading to haploinsufficiency are typically associated with milder manifestations, while glycine substitutions in the triple helix region or splicing variants are linked to a more aggressive disease course [16]. Therefore, wider monitoring intervals have been proposed for patients in the former group, while more intensive monitoring has been proposed for patients in the latter group [17]. Additionally, patients with null/haploinsufficiency variants, who are likely to have better tissue quality, may benefit from endovascular repair [18, 19]. As orthopedic surgeons, we have also explored how the integration of genotypic information with clinical manifestations can inform surgical decision-making. For example, the hypermobility of the spine may influence the selection of the lowest instrumented vertebra or the type of osteotomy in the corrective surgery for spinal deformities (data unpublished). While these advancements show significant potential, substantial work remains to be done to fully establish the effectiveness of molecular finding-guided approaches in clinical practice.

## Ongoing investigations in drug development through repurposing

Recent studies have accelerated drug development for EDS, particularly through the repurposing of existing medications to mitigate life-threatening vascular events in vEDS. Celiprolol, a beta blocker traditionally used to treat hypertension, is currently undergoing clinical trials [20]. Previous studies, including trials conducted in Europe, have provided supportive but controversial evidence regarding its efficacy in reducing the occurrence of vascular events [21]. Irbesartan, an angiotensin II receptor blocker, has recently been shown in a clinical trial to reduce the risk of arterial events when combined with the reference celiprolol treatment [22]. Enzastaurin is an oral serine/threonine kinase inhibitor originally developed for cancer treatment. Preclinical mouse studies have indicated its therapeutic potential in vEDS through modulation of the PLC/IP_3_/PKC/ERK signaling pathway [23]. Although its clinical trial has been suspended, the PLC/IP_3_/PKC/ERK axis and interconnected signaling pathways continue to attract interest, prompting the exploration of other repurposed drugs such as spironolactone, hydralazine, and sitaxentan, along with the identification of *Map2k6* as a potential protective genetic modifier [23–26].

## Future targeted therapy shaped by cutting-edge approaches

Emerging investigations using advanced technologies are uncovering mechanistic insights that pave the way for novel targeted therapies across various EDS subtypes. For example, inhibition of integrin signaling or injection of wild-type fibroblasts into Col5a1CKO mice improved poor wound healing, a hallmark feature of cEDS [27]. Inhibition of miR-29b-3p in vEDS fibroblasts has been shown to enhance the integrity of the extracellular matrix, suggesting a potential therapeutic role for miRNA inhibition [28]. In *b3galt6* knock-out zebrafish, which phenocopy spondylodysplastic EDS, a small amount of glycosaminoglycans with an immature linkage region was still produced, suggesting a potential rescue mechanism [29]. Diverse in vitro and in vivo models, such as animal models, cell lines, and patient-derived induced pluripotent stem cells, have been established to explore the etiology, pathophysiology, and pathogenesis of nearly every rare subtype of EDS, opening new possibilities for future therapeutic strategies.

As we count down to Rare Disease Day 2025, we honor the remarkable achievements in EDS research, from ending the diagnostic odyssey to exploring targeted therapies. Looking ahead, we are steadily moving toward a future where precision medicine holds the promise of long-awaited solutions for patients and families affected by EDS and other rare disorders—toward a life that is “More than You Can Imagine.”
